# IL-5 and IP-10 Detected in Quantiferon Supernatants Distinguish Latent Tuberculosis from Healthy Individuals in Areas with High Burden in Lima, Peru

**DOI:** 10.3390/pathogens14121225

**Published:** 2025-11-30

**Authors:** Nawal De la Peña Galindo, Silvia Capristano Valdez, Cesar Sanchez Neira, Henri Bailon Calderon, Gilmer Solis Sanchez, Flor Peceros Pelaez, Marco Galarza Perez

**Affiliations:** 1 Innovation and Development Area, Instituto Nacional de Salud, Lima 15046, Peru; nawal.delapena.02@unsch.edu.pe (N.D.l.P.G.); scapristano@ins.gob.pe (S.C.V.); csanchezn@ins.gob.pe (C.S.N.); hbailon@ins.gob.pe (H.B.C.); 2 Health Technology Assessment Center, Instituto Nacional de Salud, Lima 15046, Peru; gsolis@ins.gob.pe; 3 Virology Unit, Instituto Nacional de Salud, Lima 15046, Peru; fpeceros@ins.gob.pe

**Keywords:** latent tuberculosis, cytokines, chemokines, IGRAs

## Abstract

Background. Peru ranks among the countries with the highest burden of tuberculosis in Latin America. Interferon Gamma Release Assays (IGRAs), such as QuantiFERON-TB (QFT), cannot distinguish latent tuberculosis infection (LTBI) from active tuberculosis (ATB), but they provide a more specific and quantitative assessment of prior exposure to *Mycobacterium tuberculosis*. Methods. We enrolled 56 participants and measured 38 cytokines/chemokines from plasma obtained through QFT in patients with active TB (n = 11), LTBI (n = 17), and QFT-negative healthy controls (n = 28) using a Multiplex Bead Assay. Results. Levels of IL-1RA, IL-2, IL-5, IFN-γ, and IP-10 significantly differentiated both ATB and LTBI groups from healthy controls (*p* < 0.035). Furthermore, IL-5 and IP-10 specifically distinguished LTBI from healthy controls (*p* < 0.015), while GM-CSF differentiated ATB from controls (*p* < 0.05). Conclusions. Beyond IFN-γ as a differential proinflammatory cytokine in TB, cytokines such as IL-5, and IP-10 show potential as biomarkers to discriminate infection status in high-burden TB settings.

## 1. Introduction

Tuberculosis (TB) remains one of the most relevant infectious diseases worldwide, with serious implications for public health. According to World Health Organization (WHO) estimations, approximately 10.8 million people will have developed tuberculosis (TB) by 2023 worldwide. Peru’s incidence rate for the year 2023 was 173/1000 population. It is also part of the list of the 30 countries with the highest burden of MDR/RR-TB cases [1]. Lima accounts for 54.6% of TB cases in the country, with the San Juan de Lurigancho district showing the highest incidence rate in 2024 (2897 cases per 100,000 inhabitants), compared with 623 per 100,000 in Villa María del Triunfo. (http://www.tuberculosis.minsa.gob.pe/DashboardDPCTB/SalaSituacionalList.aspx). The prevalence of LTBI was estimated at 15% among medical students in 2018, and as high as 56% among healthcare workers with over 10 years of service in 2017 [2,3]. Although most individuals infected with *Mycobacterium tuberculosis* (MTB) remain asymptomatic, approximately 10% progress to active TB, representing a significant reservoir for future cases [4,5,6]. Current diagnostic methods include microscopy and immunological tests such as QuantiFERON-TB and ELISPOT assay [7]. The QFT-Plus assay measures interferon-gamma (IFN-γ) released in response to MTB-specific antigens such as ESAT-6 (Early Secreted Antigenic Target-6) and CFP-10 (Culture Filtrate Protein-10), which are found in the MTB complex, including *M. bovis*, but are absent in BCG vaccine strains and most non-tuberculous mycobacteria [8]. It is able to do so with a high sensitivity but cannot differentiate LTBI from ATB [9]. Also, the host immune system secretes cytokines and chemokines to evade bacterial invasion [10]. Cytokines play an important role in cell-mediated immune responses to MTB infection, participating in the activation, modulation, and maintenance of cellular immunity [11]. Several studies have reported the use of plasma obtained from QTF tubes for cytokine analysis [12,13,14,15,16,17,18,19,20] for its greater sensitivity and specificity in selecting Healthy Controls (HC) from patients with LTBI and ATB. Also, numerous studies show results in which the identification and quantification of cytokines and chemokines are key in the pathogenesis of TB [21,22].

This study aimed to evaluate 38 cytokines/chemokines measured in QFT-derived plasma to distinguish LTBI from ATB and compare them with healthy controls.

## 2. Materials and Methods

### 2.1. Sample Collection and Enrolled Population

**Figure 1:**
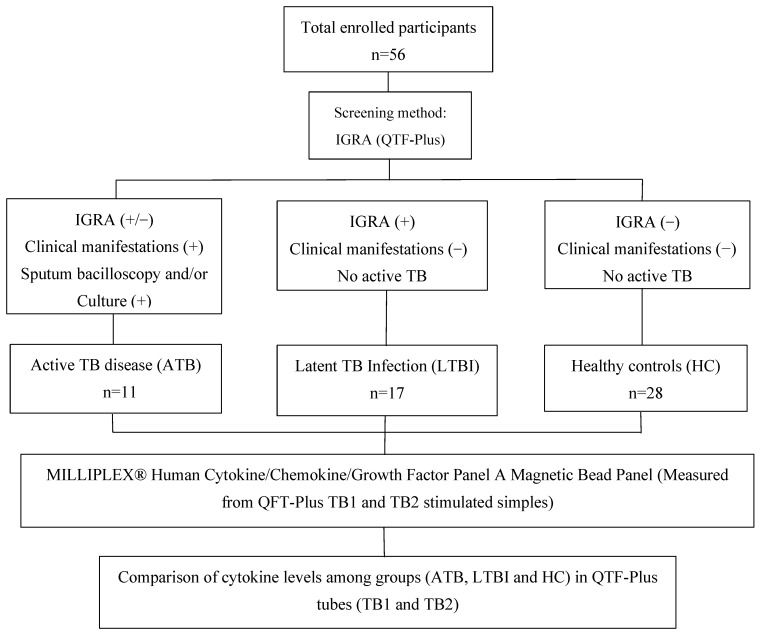
Eligibility criteria for the different participant groups included in this study.

A total of 56 participants were recruited from October 2023 to November 2024 in Lima, Peru. Samples were collected from patients at the San Juan de Lurigancho Hospital and the Villa San Luis Health Center. All participants were over 18 years of age, without autoimmune diseases or medications that could interfere with the study. Demographic and socioeconomic information was collected, including age, gender, marital status, occupation, commuting time, and BMI, as well as TB-related risk factors such as contact history and Bacille Calmette-Guérin (BCG) vaccination status. Participants were categorized into three groups based on clinical, radiological, and laboratory findings: Healthy controls (HC) were individuals with no clinical symptoms compatible with tuberculosis, a normal chest X-ray, and a negative QFT test result. Latent tuberculosis infection (LTBI) was defined as the absence of clinical or radiographic evidence of active disease, in combination with a positive QFT result. Participants in this group were asymptomatic and had negative sputum smear microscopy results. Active tuberculosis (ATB) was defined by the presence of clinical symptoms consistent with TB and radiographic findings suggestive of active pulmonary disease, confirmed by a positive sputum smear microscopy for acid-fast bacilli (AFB). All ATB participants were diagnosed prior to or at the time of sample collection and had not yet started anti-tuberculosis treatment (Figure 1). All participants provided written informed consent. The study was approved by the Institutional Committee on Ethics in Research of the Peruvian National Institute of Health (RD N° 162-2019-OGITT/INS).

### 2.2. QuantiFERON-TB GOLD In-Tube Assay

The assay was performed according to the manufacturer’s instructions. One milliliter of whole blood was added to each of the four QFT tubes (TB1, TB2, mitogen control, and negative control). After incubation at 37 °C for 24 h, tubes were centrifuged and plasma stored at −20 °C. Interferon-gamma (IFN-γ) was quantified by ELISA. Excess plasma was frozen and stored at −80 °C for cytokine/chemokine analysis.

### 2.3. Multiplex Cytokine Analysis

Plasma cytokine/chemokine concentrations were measured using a Millipore Milliplex map system IFU-HCYTA-60K (EMD Millipore Corporation, Billerica, MA, USA). Thirty-eight cytokines/chemokines were analyzed in this study (Table 1). For multiplex bead assays, the manufacturer’s protocol was followed: 25 μL of standard and 25 μL of plasma along with 25 μL of mixed beads were added to the corresponding wells; 25 μL of matrix solution and 25 μL of assay buffer were added separately to the standard wells and sample wells. After 16 h of incubation at 2–8 °C with constant shaking, followed by washing, detection antibodies were added to the wells and incubated for 1h at room temperature, followed by washing. Finally, 25 μL of streptavidin-phycoerythrin was added per well and incubated for 30 min at room temperature. The contents of the wells were then removed and washed with a wash buffer. The mixed microspheres were re-suspended with 150 μL of Sheath Fluid Plus. Data were collected and analyzed using LIFECODES Fluoroanalyzer equipment ((Immucor GTI Diagnostics, Inc., Waukesha, WI, USA) and Milliplex Analyst software version 5.1 (5.1.0.0) (EMD Millipore Corporation, Billerica, MA, USA)).

### 2.4. Statistical Analysis

Statistical analyses were performed using Stata V17.0 (Stata Corporation, College Station, TX, USA) and GraphPad Prism version 10.5.0 (GraphPad Software, San Diego, CA, USA). The Shapiro–Wilk test assessed normality. Depending on data distribution, results were expressed as mean ± SD or median (IQR). Comparisons among groups were performed using the Kruskal–Wallis test with Bonferroni correction. Values below zero were excluded. Heatmaps for log2-transformed cytokine levels were generated to assess group clustering.

## 3. Results

### 3.1. Sociodemographic Characteristics of Study Participants

Of a total of 56 participants, 62.5% were women and of these 6 (17.1%), 12 (34.3%) and 17 (48.6%) were HC, LTBI and ATB, respectively. The average age of HC was 37.8 years, while that of patients with LTBI was 47.1 and ATB was 42.6 years. A 30.4% (17/56) presented normal BMI, 48.2% (27/56) overweight, 17.9% (10/56) class I obesity and 3.6% (2/56) class II obesity. About 16.1% indicated that they had previously tested negative on a tuberculin skin test (TST). Also, 89.3% declared that they had been vaccinated with BCG. Among the different modes of transportation, buses (64.3%) were the most used for travel. In addition, 76.8% reported having been in contact with a patient with TB (Supplementary Table S1).

In the analysis of the demographic characteristics of the participants (Table 2), transportation travel time showed significant differences between the groups. Patients with ATB and LTBI reported travel times of less than one hour, while HC patients reported longer travel times (*p* = 0.0313). Also, the QTF test results showed very significant differences between the groups. HC participants had negative results, while the ATB and LTBI groups had a higher proportion of positive or, in some cases, indeterminate results (*p* < 0.0001). No statistically significant differences were observed between the groups in terms of gender (*p* = 0.6671), age (*p* = 0.5524), ethnicity (*p* = 0.6909), marital status (*p* = 0.3711), occupation (*p* = 0.528) or nutritional status assessed by body mass index (BMI) (*p* = 0.6291). Finally, BCG vaccination status, no significant differences were found (*p* = 0.5749), although most participants in all groups reported having received the vaccine.

### 3.2. Plasma Cytokine Profile and Its Dynamic Response to Change in Tuberculosis Infection

At first observation, the thirty-eight cytokines/chemokines analyzed were measured correctly in each group. Some cytokines such as EGF, VEGF, IL3- and IL-7 were less measurable in tubes TB1 and TB2 compared to all the others because when their respective Nil value was subtracted, the result was negative and was considered missing data.

**Figure 2:**
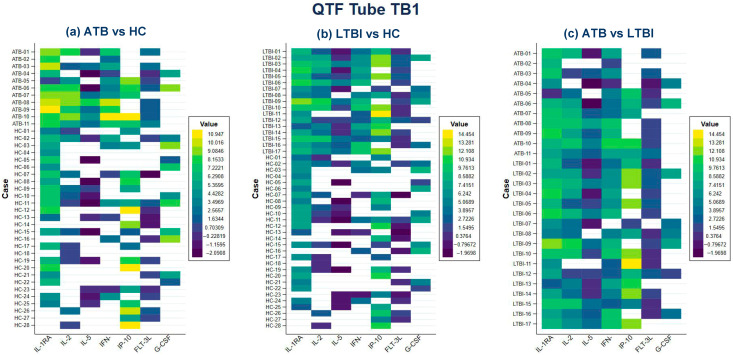
Heatmaps representing cytokine/chemokine measurements by log 2 conversion in QTF Tube TB1 comparing (**a**) ATB vs. HC, (**b**) LTBI vs. HC and (**c**) ATB vs. LTBI. High levels of cytokines are indicated in yellow and low levels of cytokines are indicated in blue, the white boxes are missing data.

In the TB1 tube, the levels of FLT-3L, GMCSF, IFN-γ, IL-1A, IL-1RA, IL-2, IL-3, IL-5, IL-8, IL-17A, IP-10, MCP-1 and MCP-3 in LTBI patients were increased compared to HC. Similarly, cytokines CD40L, EGF, EOTAXIN, FGF2, Fractalkine, GCSF, GRO, IFNA2, IL-12P70 and VEGFA were decreased compared to HC. In addition, the levels of FLT-3L, GCSF, GMCSF, IFN-γ, IL-1RA, IL-2, IL-4, IL-5, IL-8, IL-12P40, IL-13, IL-17A, IP-10, MCP-3 and MDC in ATB patients were increased compared to HC while levels of CD40L, EGF, IL-10, MCP-1, MIP1A and VEGFA were decreased. Notably, the levels of cytokines IL-1RA, IL-2, IL-5, G-CSF, IFN-γ, IP-10, and FLT-3L showed significant differences among three groups. (Supplementary Table S2 and Figure 2a–c).

Heatmap analysis showed that the expression profiles of IL-1RA, IL-2 and IP-10 had increased values when comparing ATB vs. HC (Figure 2a). However, when comparing LTBI vs. HC, only IL-1RA and IP-10 were increased (Figure 2b). In contrast, when comparing ATB vs. LTBI, decreased concentrations of IP-10 were observed (Figure 2c).

**Figure 3:**
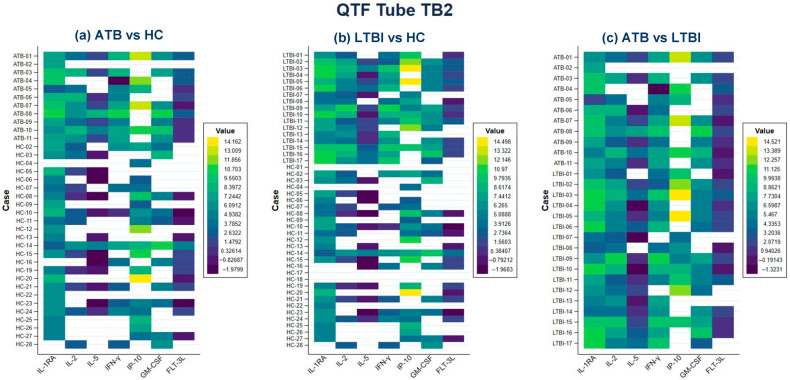
Heatmaps representing cytokine/chemokine measurements by log 2 conversion in QTF Tube TB2 comparing (**a**) ATB vs. HC, (**b**) LTBI vs. HC and (**c**) ATB vs. LTBI. High levels of cytokines are indicated in yellow and low levels of cytokines are indicated in blue, the white boxes are missing data.

In the TB2 tube, the levels of GMCSF, IFN-γ, IL-1RA, IL-2, IL-3, IL-5, IL-8, IP-10 and MIP1B in LTBI patients were increased compared to HC. Similarly, cytokines CD40L, EGF, FGF2, Fractalkine, GCSF, GRO, IFNA2, IL-1A, IL-6, IL-12P70, MCP-1, TGFA and TNFB were decreased compared to HC. In addition, the levels of Fractalkine, GCSF, GMCSF, IFN-γ, IL-1B, IL-1RA, IL-2, IL-5, IL-8, IL-12P40, IL-13, IP-10, MCP-3 and TNFA in ATB patients were increased compared to HC while levels of EGF, GRO, IL-1A, IL-6, MCP-1 and MIP1A were decreased. Also, the levels of cytokines GMCSF, IFN-γ, IL-1RA, IL-2, IL-5 and IP-10 showed significant differences among three groups (Supplementary Table S3 and Figure 3a–c). Heatmap analysis showed that the expression profiles of IL-1RA, IL-2, IFN-γ and IP-10 had increased values when comparing ATB and LTBI vs. HC (Figure 3a,b). In addition, when comparing ATB vs. LTBI, decreased concentrations of IL-1RA and IFN-γ were observed while IP-10 was increased (Figure 3c).

### 3.3. Identification of Cytokine to Distinguish ATB and LTBI Subjects from Healthy Controls

**Figure 4:**
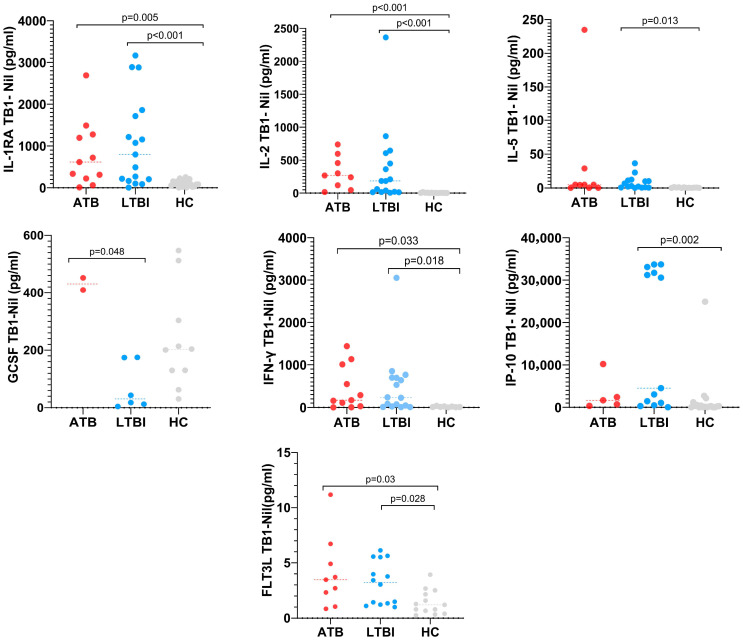
Differential abundance values of cytokines/chemokines comparing ATB and LTBI cases with HC group in QFT-Plus TB1 antigen tubes. Horizontal lines indicate statistically significant differences between groups (*p* < 0.05).

In the TB1 tube, IL-1RA levels were 10-fold higher in patients with LTBI and 7-fold higher in patients with ATB than in HC. Statistical significance analysis showed that this cytokine can distinguish between LTBI and HC (*p* < 0.001) as well as between ATB and HC (*p* = 0.005). IL-2 showed a similar pattern, with levels 90-fold higher in LTBI and more than 70-fold higher in ATB compared to HC. Statistical analysis showed a *p* < 0.001 in both cases. On the other hand, IL-5 level was 16-fold higher in patients with LTBI than in HC (*p* = 0.013). GCSF level was 6-fold higher in patients with ATB than in LTBI with *p* = 0.048. IFN-γ levels were 33-fold higher in patients with LTBI and 29-fold higher in patients with ATB than in HC. Statistical significance analysis showed that this cytokine can distinguish between LTBI and HC (*p* < 0.033) as well as between ATB and HC (*p* = 0.018). In addition, IP-10 level was 8-fold higher in patients with LTBI than in HC (*p* = 0.002). Similarly, FLT3L levels were 2-fold higher in patients with LTBI and 2.5-fold higher in patients with ATB than in HC. Statistical significance analysis showed that this cytokine can distinguish between LTBI and HC (*p* = 0.028) as well as between ATB and HC (*p* = 0.03) (Figure 4).

**Figure 5:**
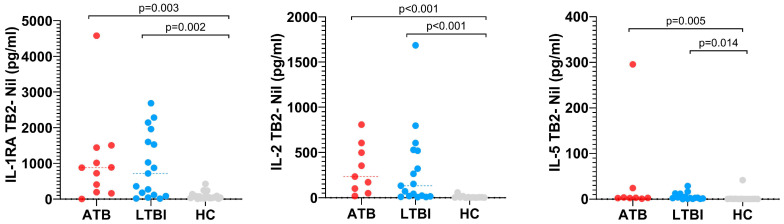

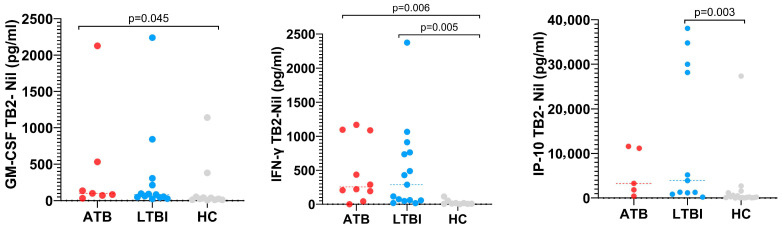
Cytokine/chemokine abundances showing significant differences between ATB, LTBI and HC groups in QFT-Plus TB2 antigen tubes. Statistically significant differences in cytokine levels between the study groups are indicated by horizontal lines (*p* < 0.05).

In the TB2 tube, IL-1RA levels were 10-fold higher in patients with LTBI and 11-fold higher in patients with ATB than in HC. Statistical significance analysis showed that this cytokine can distinguish between LTBI and HC (*p* = 0.002) as well as between ATB and HC (*p* = 0.003). IL-2 showed a similar pattern, with levels 33-fold higher in LTBI and more than 35-fold higher in ATB compared to HC. Statistical analysis showed a *p* < 0.001 in both cases. On the other hand, IL-5 level was 2-fold higher in LTBI and 10-fold higher in ATB compared to HC. Statistical significance analysis showed that this cytokine can distinguish between LTBI and HC (*p* = 0.014) as well as between ATB and HC (*p* = 0.005). GM-CSF level was 2.5-fold higher in ATB in comparison to HC. This cytokine was able to distinguish between ATB and HC with *p* = 0.045. In addition, IFN-γ levels were 18-fold higher in patients with LTBI and ATB. Statistical significance analysis showed that this cytokine can distinguish between LTBI and HC (*p* < 0.005) as well as between ATB and HC (*p* = 0.006). Similarly, IP-10 level was 6-fold higher in patients with LTBI than in HC (*p* = 0.003) (Figure 5).

### 3.4. Accuracy of Each Cytokine Marker in the QFT TB1 and TB2 Tubes for Discriminating Between LTBI and HC Groups

To elucidate the accuracy of these markers in the diagnosis of LTBI, sensitivities and specificities were calculated using the value with the highest Youden index as the cutoff value. The cut-off values of the six cytokines in distinguishing between LTBI and HC in the TB1 tube were 1.36 pg/mL (IL-5), 3.04 pg/mL (FLT-3L), 12.39 pg/mL (IL-2), 37.25 pg/mL (IFN-γ), 203.91 pg/mL (IL-1RA) and 485.79 pg/mL (IP-10), respectively. IL-2 had the highest sensitivity (93.0%) and specificity (91.0%) while FLT-3L had the lower sensitivity (57.0%) but acceptable specificity (92.0%) among the six cytokines. On the other hand, in TB2 tube, the cut-off values of the five cytokines were 1.46 pg/mL (IL-5), 57.15 pg/mL (IFN-γ), 859.31 pg/mL (IP-10), 8.04 pg/mL (IL-2) and 270.9 pg/mL (IL-1RA), respectively. In this tube, the IP10 value was almost double that in TB1. IL-2 had the highest sensitivity (94.0%) while IL-1RA had the highest specificity (95.0%) (Table 3).

### 3.5. Accuracy of Each Cytokine Marker in the QFT TB1 and TB2 Tubes for Discriminating Between ATB and HC Groups

In the TB1 tube, the cut-off values of the four cytokines in distinguishing between ATB and HC in the TB1 tube were 29.13 pg/mL (IFN-γ), 2.32 pg/mL (FLT-3L), 15.93 pg/mL (IL-2) and 221.65 pg/mL (IL-1RA). IL-2 had the highest sensitivity (100.0%) and specificity (91.0%) while FLT-3L had the lower sensitivity (78.0%) and specificity (76.0%). In addition, in the TB2 tube, the cut-off values of the five cytokines were 71.32 pg/mL (GM-CSF), 2.21 pg/mL (IL-5), 114.15 pg/mL (IFN-γ), 49.65 pg/mL (IL-2) and 161.73 pg/mL (IL-1RA) (Table 4).

## 4. Discussion

The identification of individuals with latent tuberculosis infection (LTBI) and the prompt diagnosis of active tuberculosis (ATB) are essential to initiate early treatment and reduce disease transmission. IGRAs, such as QFT, cannot differentiate between ATB and LTBI, but the increased sensitivity and specificity of these tests for diagnosing MTB infection is well recognized.

Research into biomarkers for TB is crucial to achieving global goals for eliminating the disease. Efforts to understand immunological changes, such as cytokines and their differential expression during the different stages of TB are valuable for identifying unique biological signatures.

In this study, we evaluated the diagnostic efficacy of plasma cytokines in diagnosing the stages of MTB infection (LTBI and ATB) in adult Peruvian patients. The results showed that plasma cytokine secretion characteristics can differentiate between the ATB, LTBI, and HC groups. Among the 38 cytokines analyzed, IL-1RA, IL-2, IL-5, IFN-γ, IP-10, GM-CSF, G-CSF, and FLT-3L exhibited differential expression patterns between ATB, LTBI, and healthy controls.

IL-2 emerged as the most robust biomarker of ATB and LTBI with adequate sensitivity and specificity in both TB1 and TB2 tubes. To distinguish between the LBTI group, the sensitivity and specificity values for tube TB1 were 93.0% and 91.0%, respectively, while for tube TB2, these values were 94.0% and 83.0%, respectively. In contrast, to distinguish between the ATB group, the sensitivity and specificity values for tube TB1 were 100.0% and 91.0%, respectively, while for tube TB2, these values were 89.0% and 92.0%, respectively. This is consistent with previous findings suggesting that IL-2 is an indicator of LTBI and ATB due to its association with central memory T cell responses [23]. In addition, the chemokine IP-10 to distinguish between the LTBI group, the sensitivity and specificity values for tube TB1 were 84.0% and 72.0%, respectively, while for tube TB2, these values were 93.0% and 76.0%, respectively. IP-10 (CXCL10) is induced by IFN-γ and is known to recruit T cells to sites of infection. Elevated levels in LTBI may reflect persistent immune activation without overt disease, supporting its potential use as an additional biomarker to IFN-γ in addition to IGRA [14,24].

Similarly, IL-1RA showed potential as a biomarker that can distinguish between LTBI and ATB with respect to HC. To distinguish between the LBTI group, the sensitivity and specificity values for tube TB1 were 76.0% and 85.0%, respectively, while for tube TB2, these values were 64.0% and 95.0%, respectively. In contrast, to distinguish between the ATB group, the sensitivity and specificity values for tube TB1 were 82.0% and 90.0%, respectively, while for tube TB2, these values were 91.0% and 82.0%, respectively. These findings corroborate previous research and reinforce the value of multi-cytokine panels for improving TB staging. IL-1RA, an anti-inflammatory cytokine secreted by monocytes and epithelial cells, is expressed at elevated levels in both LTBI and ATB, suggesting a compensatory response to inflammation. Notably, previous studies have shown that IL-1RA levels decrease following TB treatment, supporting its potential role as a biomarker for disease activity. However, due to variability in expression, optimized assays are needed for its diagnostic application [25].

Similarly, the cytokines FLT-3L and GM-CSF showed acceptable sensitivities and specificities for distinguishing between LTBI and ATB with respect to HC. To distinguish LTBI, FLT-3L showed a sensitivity of 57.0% and specificity of 92.0% in tube TB1, while to distinguish ATB the sensitivity and specificity were 78.0% and 76.0%, respectively, also only in the TB1 tube. GM-CSF was able to distinguish between ATB and HC showing a sensitivity of 86.0% and specificity of 78.0% in tube TB2. FLT3L, while less frequently studied in TB, showed promise as a specific biomarker in TB1 tubes, with strong specificity but moderate sensitivity. FLT3L is involved in dendritic cell proliferation and may reflect the expansion of antigen-presenting cells during infection [26]. GM-CSF is a cytokine involved in the activation and differentiation of macrophages. Elevated levels of GM-CSF in ATB may indicate increased recruitment and activation of myeloid cells during the active phase of the disease. Interestingly, GM-CSF could differentiate TB from HC and, in some cases, from LTBI. This is consistent with recent studies suggesting that GM-CSF contributes to the host’s defense against MTB and may be a therapeutic target or vaccine adjuvant [27]. Interestingly, G-CSF was the only cytokine that showed statistical significance in distinguishing between ATB and LTBI only in the TB1 tube.

IFN-γ remains a key cytokine in IGRA-based assays. Its performance in our study was consistent with its known role in TB immunopathogenesis. Its elevated expression in both TB and LTBI compared to HC reflects the intensified cellular immune response induced by MTB-specific antigens [6,7]. However, IFN-γ alone is insufficient to discriminate between TB and LTBI. Therefore, its combination with other cytokines, such as IL-2 and IL-1RA, improves the diagnostic accuracy of the disease [28].

Similarly, to distinguish between the LBTI and HC groups, the sensitivity and specificity values for IL-5 in tube TB1 were 71.0% and 90.0%, respectively, while for tube TB2, these values were 84.0% and 90.0%, respectively. In contrast, to distinguish between the ATB and HC groups, the sensitivity and specificity values for tube TB2 were 88.0% and 91.0%, respectively. IL-5 is a Th2-associated cytokine involved in B-cell activation and eosinophil differentiation. The higher concentration of IL-5 in LTBI may reflect a less Th1-polarized or more regulated immune profile that helps contain inflammation and prevent tissue damage. In contrast, during ATB, the predominant Th1-type response (characterized by IFN-γ and TNF-α production) and sustained inflammation may suppress Th2 responses detectable in stimulated assays. Alternatively, elevated IL-5 levels in LTBI might represent a protective or host-control signature against MTB replication. These findings suggest heterogeneity in the immune response to MTB and highlight the need for longitudinal studies to determine whether IL-5 could serve as a biomarker of immune regulation or protection against progression to ATB disease [29].

Although several of the cytokines analyzed in this study did not show statistically significant differences between groups, previous research has identified them as potential biomarkers in the pathogenesis of TB. Suzukawa et al. demonstrated that the combined measurement of IFN-γ, IL-2, IL-5, IL-10, IL-1RA, and MCP-1 in QuantiFERON assay supernatants was useful in distinguishing ATB from LTBI [16].

In addition, recent studies have shown that specific cytokine profiles can predict clinical outcomes. Pandiarajan et al. reported that levels of IL-2, IFN-γ, TNF-α, IL-6, IL-10, IL-17A, IL-18, and GM-CSF before treatment and two months after antigen stimulation differed significantly between patients with unfavorable outcomes and those with favorable outcomes. -6, IL-10, IL-17A, IL-18, and GM-CSF before treatment and two months after antigen stimulation differed significantly between patients with unfavorable outcomes and those who achieved cure, with notable ROC values for IL-10, IL-17A, and TNF-α [30]. Complementary, Rajamanickam et al. reported that GM-CSF, IP-10, and IL-1RA may help identify TB contacts at high risk of progression [31]. In our study, IP-10 concentrations were higher in individuals with LTBI compared with those with ATB. IP-10 (CXCL10) is a chemokine induced by IFN-γ that promotes the recruitment of activated Th1 lymphocytes and monocytes to sites of MTB infection. Elevated IP-10 levels in LTBI may reflect a controlled, yet sustained, immune activation capable of containing bacillary replication without causing overt pathology. This pattern is consistent with the notion that LTBI represents a state of immune equilibrium, in which continuous antigenic stimulation maintains IFN-γ–induced chemokines such as IP-10. In contrast, during ATB disease, excessive inflammation and immune exhaustion can alter cytokine and chemokine profiles, potentially resulting in lower IP-10 production in stimulated assays. Therefore, increased IP-10 in LTBI might represent an immune signature of effective containment rather than active tissue damage, supporting its potential use as a biomarker for infection control or latency [32,33]. Similarly, previous studies support the usefulness of IP-10 as an immunological biomarker in the diagnosis of TB (Supplementary Table S4).

Other studies have described predictive signatures; for example, a combination of IL-8, IL-10, and CCL3 accurately predicted TB progression in close contacts [34]. Finally, it has been suggested that GM-CSF, VEGF, IL-3, IP-10, IL-10, and IL-9 are early biomarkers of TB development risk [35].

The search for biomarkers that can distinguish between TB and LTBI is an ongoing task for various research groups around the world. In Latin America, there is little research on the immune response to MTB infection. This study is the second after Guio et al., who used other different mycobacterial antigens [36]. Most related studies correspond to African or Asian populations. Local epidemiological factors, BCG vaccination rates, and environmental exposure to mycobacteria can influence baseline cytokine responses, making it necessary to validate specific biomarkers for each region [9,11].

Limitations include the relatively small ATB sample size and the restriction to 38 cytokines. Future studies should validate these results in larger and more diverse cohorts, including pediatric and immunocompromised populations. Nonetheless, the present findings provide valuable insight into MTB immunopathogenesis in Peru.

Our findings align with prior research indicating that multi-cytokine panels improve diagnostic accuracy beyond IFN-γ alone. This is particularly relevant in Latin American populations, where few studies have explored host immune responses to TB. Despite the limited sample size, our results suggest that incorporating IL-5 and IP-10 into diagnostic platforms could strengthen TB control strategies in Peru. In addition, these findings suggest that the evaluated biomarkers could serve as useful tools for improving TB screening strategies, particularly among migrant populations, who often present challenges for timely diagnosis and follow-up [37]. Incorporating these biomarkers into existing screening programs could enhance the identification of LTBI and ATB infections, allowing earlier intervention and reducing transmission risk. Therefore, their potential application in migrant health programs represents an important step toward strengthening TB control and advancing global public health goals.

## 5. Conclusions

In conclusion, cytokine profiling of QFT-Plus supernatants demonstrated that IL-5 and IP-10 can reliably distinguish LTBI from healthy individuals, while IL-2 and GM-CSF differentiate ATB from controls. These biomarkers, integrated into immunological platforms, could significantly improve TB diagnosis and patient management in High-Burden settings such as Peru. Moreover, their incorporation into screening programs, particularly for migrant populations, could enhance early detection and strengthen TB control efforts within Public Health Systems.

## Figures and Tables

**Table 1:** List of 38 cytokines/chemokines used in this study and their function.

Type	Cytokines/Chemokines
Proinflammatory cytokines	CD40L, IFN-α, IFN-γ, IL-1α, IL-1β, IL-3, IL-5, IL-6, IL-9, IL-12 (p40), IL-12 (p70), IL-17α, TNF-α, TNF-β
Anti-inflammatory cytokines	IL-1RA, IL-4, IL-10, IL-13
Chemokines	Eotaxin, Fractalkine, GRO, IL-8, IP-10, MCP-1, MCP-3, MDC, MIP-1α, MIP-1β
Growth factors	EGF, FGF-2, Flt-3L, G-CSF, GM-CSF, IL-2, IL-3, IL-15, IL-7, VEGFA

**Table 2:** Clinical Characteristics of 56 participants of this study.

		ATB (%)	LTBI (%)	HC (%)	*p* Value
		(n = 11)	(n = 17)	(n = 28)	
Gender	Female	6 (17.1)	12 (34.3)	17 (48.6)	0.6671 †
	Male	5 (23.8)	5 (23.8)	11 (52.4)	
Age (years)	22–29	2 (22.2)	1 (11.1)	6 (66.7)	0.5524 ‡
	30–37	2 (14.3)	3 (21.4)	9 (64.3)	
	38–45	3 (20.0)	5 (33.3)	7 (46.7)	
	46 or more	4 (22.2)	8 (44.4)	6 (33.3)	
Ethnicity	Mestizo	11 (20.4)	17 (31.5)	26 (48.1)	0.6909 ‡
	White	0 (0.0)	0 (0.0)	2 (100.0)	
Marital status	Married	5 (27.8)	7 (38.9)	6 (33.3)	0.3711 ‡
	Single	3 (15.0)	3 (15.0)	14 (70.0)	
	Cohabiting	3 (21.4)	5 (35.7)	6 (42.9)	
	Separated	0 (0.0)	1 (50.0)	1 (50.0)	
	Divorced	0 (0.0)	0 (0.0)	1 (100.0)	
	Widow	0 (0.0)	1 (100.0)	0 (0.0)	
Working type	Dependent	4 (13.8)	8 (27.6)	17 (58.6)	0.528 ‡
	Independent	3 (18.8)	6 (37.5)	7 (43.8)	
	House	2 (33.3)	2 (33.3)	2 (33.3)	
	Student	1 (33.3)	0 (0.0)	2 (66.7)	
	Eventual	1 (50.0)	1 (50.0)	0 (0.0)	
Travel time by transport	Less than one hour	6 (22.2)	11 (40.7)	10 (37.0)	0.0313 ‡
	One hour	3 (18.8)	2 (12.5)	11 (68.8)	
	Two hours	0 (0.0)	2 (22.2)	7 (77.8)	
	Three hours or more	2 (50.0)	2 (50.0)	0 (0.0)	
BCG Vaccine	Yes	9 (17.6)	16 (31.4)	26 (51.0)	0.5749 ‡
	No	2 (40.0)	1 (20.0)	2 (40.0)	
QTF assay result	Positive	7 (29.2)	17 (70.8)	0 (0.0)	<0.0001 ‡
	Negative	1 (3.4)	0 (0.0)	28 (96.6)	
	Indeterminate	3 (100.0)	0 (0.0)	0 (0.0)	
BMI	Normal	6 (35.3)	5 (29.4)	6 (35.3)	0.6291 ‡
	Overweight	4 (14.8)	8 (29.6)	15 (55.6)	
	Class I obesity	1 (10.0)	3 (30.0)	6 (60.0)	
	Class II obesity	0 (0.0)	1 (50.0)	1 (50.0)	

ATB: Active TB; LTBI: Latent TB; HC: Health control; QTF assay: Quantiferon assay; BMI: Body Mass Index; † Pearson’s Chi Square Test. ‡ Fisher’s Exact Test.

**Table 3:** Diagnostic performance of individual biomarkers to MTB-specific antigens in discriminating between LTBI and HC groups.

Tube	Marker	Cut-Off (pg/mL)	Sensitivity (95% CI)	Specificity (95% CI)
TB1-Nil	IFN-g	37.25	75 (54–96)	87 (64–100)
	IL-5	1.36	71 (47–95)	90 (71–100)
	IP-10	485.79	84 (65–100)	72 (51–92)
	FLT-3L	3.04	57 (31–83)	92 (78–100)
	IL-2	12.39	93 (81–100)	91 (76–100)
	IL-1RA	203.91	76 (56–96)	85 (70–100)
TB2-Nil	IL-5	1.46	84 (65–100)	90 (73–100)
	IFN-g	57.15	80 (59–100)	88 (68–100)
	IP-10	859.31	93 (73–100)	76 (56–97)
	IL-2	8.04	94 (83–100)	83 (62–100)
	IL-1RA	270.9	64 (41–87)	95 (86–100)

TB1: Quantiferon test tube 1 stimulated with MTB antigens; TB2: Quantiferon test tube 2 stimulated with MTB antigens; CI: Confidence interval.

**Table 4:** Diagnostic performance of individual biomarkers to MTB-specific antigens in discriminating between ATB and HC groups.

Tube	Marker	Cut-Off (pg/mL)	Sensitivity (95% CI)	Specificity (95% CI)
TB1-Nil	IFN-g	29.13	82 (59–100)	87 (64–100)
	FLT-3L	2.32	78 (50–100)	76 (54–100)
	IL-2	15.93	100 (100–100)	91 (76–100)
	IL-1RA	221.65	82 (59–100)	90 (78–100)
TB2-Nil	GM-CSF	71.32	86 (60–100)	78 (51–100)
	IL-5	2.21	88 (65–100)	91 (74–100)
	IFN-g	114.15	80 (55–100)	89 (68–100)
	IL-2	49.65	89 (68–100)	92 (76–100)
	IL-1RA	161.73	91 (73–100)	82 (66–98)

TB1: Quantiferon test tube 1 stimulated with MTB antigens; TB2: Quantiferon test tube 2 stimulated with MTB antigens; CI: Confidence interval.

## Data Availability

The data presented in this study are available on request from the corresponding author.

## Supplementary Materials

The following supporting information can be downloaded at: https://www.mdpi.com/article/10.3390/pathogens14121225/s1, Supplementary Table S1: Individual patient data listing in this study; Supplementary Table S2: Cytokine concentration in QFT-Plus TB1 tube from patients with LTBI, ATB and HC; Supplementary Table S3: Cytokine concentration in QFT-Plus TB2 tube from patients with LTBI, ATB and HC; Supplementary Table S4: Studies related to IP-10 and Tuberculosis.
