# Supplementary material for: IL-5 and IP-10 Detected in Quantiferon Supernatants Distinguish Latent Tuberculosis from Healthy Individuals in Areas with High Burden in Lima, Peru
Source: Pathogens. 2025 Nov 30;14(12):1225. doi: 10.3390/pathogens14121225 (PMC12735898; doi:10.3390/pathogens14121225)
Supplement: Supplementary file 1 [file pathogens-14-01225-s001.zip › pathogens-3919716-supplementary.pdf]

**Supplementary Table S1**. Individual patient data listing in this study.

| Samples | Participants | Origin | Gender | Age | BMI | Classification BMI | Previous TST result | Vaccine BCG | Occupation | People living at home | Type of transport | Time of employment to move | Contact with a TB patient |
|---|---|---|---|---|---|---|---|---|---|---|---|---|---|
| 1 | HC | VSL | F | 31 | 31.96 | Class I obesity | ND | Yes | Student | 4 | Bus | Two hours | Yes |
| 2 | HC | VSL | F | 33 | 20.57 | Normal | Negative | Yes | Dependent | 2 | Own car | Two hours | Yes |
| 3 | HC | VSL | M | 43 | 23.88 | Normal | ND | Yes | Dependent | 8 | particular | One hour | Yes |
| 4 | HC | VSL | M | 33 | 24.28 | Normal | Negative | Yes | Dependent | 1 | On foot | Less than an hour | Yes |
| 5 | HC | HSJL | F | 39 | 29.14 | Overweight | Negative | Yes | Dependent | 4 | Train | Less than an hour | Not |
| 6 | HC | HSJL | F | 46 | 28.00 | Overweight | Negative | Yes | Dependent | 3 | Bus | One hour | Yes |
| 7 | HC | VSL | F | 38 | 36.94 | Class II obesity | Negative | Yes | Dependent | 4 | Train | One hour | Yes |
| 8 | HC | VSL | F | 36 | 26.56 | Overweight | ND | Yes | Dependent | 2 | Bus | Two hours | Yes |
| 9 | HC | HSJL | F | 30 | 24.89 | Normal | ND | Yes | Dependent | 4 | Bus | Two hours | Yes |
| 10 | HC | HSJL | M | 52 | 27.25 | Overweight | Negative | Yes | Dependent | 1 | Train | One hour | Not |
| 11 | HC | HSJL | F | 46 | 25.97 | Overweight | ND | Yes | Dependent | 1 | Bus | One hour | Yes |
| 12 | HC | HSJL | M | 39 | 27.89 | Overweight | ND | Yes | Dependent | 5 | Own car | Less than an hour | Yes |
| 13 | HC | HSJL | M | 25 | 24.09 | Normal | ND | Yes | Dependent | 4 | Bus | One hour | Yes |
| 14 | HC | HSJL | M | 47 | 26.37 | Overweight | ND | Yes | Dependent | 4 | Mototaxi | Two hours | Yes |
| 15 | HC | HSJL | F | 36 | 27.94 | Overweight | ND | Yes | Independent | 5 | Bus | Less than an hour | Yes |
| 16 | HC | HSJL | F | 36 | 30.04 | Class I obesity | ND | Yes | Independent | 4 | Bus | One hour | Yes |
| 17 | HC | HSJL | F | 40 | 30.41 | Class I obesity | ND | Yes | Housewife | 8 | Bus | One hour | Yes |
| 18 | HC | HSJL | F | 27 | 25.97 | Overweight | ND | Yes | Independent | 14 | Bus | One hour | Yes |
| 19 | HC | HSJL | F | 59 | 27.51 | Overweight | ND | Yes | Independent | 3 | Bus | One hour | Yes |
| 20 | HC | HSJL | F | 34 | 32.87 | Class I obesity | ND | Not | Independent | 7 | Bus | Less than an hour | Yes |
| 21 | HC | HSJL | F | 29 | 25.91 | Overweight | ND | Yes | Independent | 7 | Bus | Less than an hour | Yes |
| 22 | HC | HSJL | M | 51 | 26.71 | Overweight | ND | Yes | Dependent | 5 | Mototaxi | Less than an hour | Yes |
| 23 | HC | HSJL | F | 27 | 28.51 | Overweight | ND | Yes | Dependent | 5 | Bus | Less than an hour | Yes |
| 24 | HC | HSJL | M | 33 | 31.49 | Class I obesity | ND | Yes | Independent | 5 | Own car | One hour | Yes |
| 25 | HC | VSL | M | 27 | 25.56 | Overweight | ND | Yes | Student | 4 | Bus | Less than an hour | Yes |
| 26 | HC | VSL | F | 44 | 20.50 | Normal | ND | Yes | Housewife | 3 | Bus | Less than an hour | Yes |
| 27 | HC | VSL | M | 28 | 27.10 | Overweight | ND | Yes | Dependent | 4 | Bus | Two hours | Not |
| 28 | HC | VSL | M | 48 | 30.12 | Class I obesity | Negative | Not | Dependent | 4 | Own car | Two hours | Not |
| 29 | LTBI | VSL | F | 37 | 20.93 | Normal | ND | Yes | Dependent | 2 | Bus | Four hours | Not |
| 30 | LTBI | HSJL | F | 58 | 20.31 | Normal | Positive | Yes | Dependent | 4 | Bus | Less than an hour | Yes |
| 31 | LTBI | HSJL | F | 40 | 29.30 | Overweight | Positive | Yes | Dependent | 4 | Bus | Less than an hour | Yes |
| 32 | LTBI | VSL | M | 50 | 38.72 | Class II obesity | Positive | Yes | Dependent | 11 | Bus | Two hours | Yes |
| 33 | LTBI | HSJL | F | 65 | 26.56 | Overweight | Positive | Yes | Dependent | 2 | On foot | Less than an hour | Not |
| 34 | LTBI | HSJL | M | 48 | 26.99 | Overweight | ND | Yes | Independent | 5 | Bus | One hour | Not |
| 35 | LTBI | HSJL | F | 50 | 24.89 | Normal | ND | Yes | Dependent | 3 | On foot | Less than an hour | Yes |
| 36 | LTBI | HSJL | M | 55 | 28.41 | Overweight | ND | Yes | Independent | 3 | Bus | Less than an hour | Yes |
| 37 | LTBI | HSJL | F | 42 | 23.74 | Normal | Negative | Yes | Dependent | 3 | Bus | Less than an hour | Yes |
| 38 | LTBI | HSJL | F | 62 | 34.63 | Class I obesity | ND | Not | Independent | 6 | Mototaxi | Less than an hour | Yes |
| 39 | LTBI | HSJL | M | 33 | 32.21 | Class I obesity | ND | Yes | Dependent | 7 | Bus | Less than an hour | Yes |
| 40 | LTBI | HSJL | F | 65 | 33.29 | Class I obesity | ND | Yes | Independent | 4 | Bus | Two hours | Yes |

| | | | | | | | | | | | | | |
|---|---|---|---|---|---|---|---|---|---|---|---|---|---|
| 41 | LTBI | HSJL | F | 42 | 27.11 | Overweight | Positive | Not | Housewife | 10 | Bus | Less than an hour | Yes |
| 42 | LTBI | HSJL | M | 45 | 28.96 | Overweight | ND | Yes | Independent | 6 | Mototaxi | Four hours | Yes |
| 43 | LTBI | VSL | F | 35 | 27.43 | Overweight | ND | Yes | Independent | 7 | Mototaxi | Less than an hour | Yes |
| 44 | LTBI | VSL | F | 42 | 19.71 | Normal | ND | Yes | Casual labor | 5 | Bus | One hour | Yes |
| 45 | LTBI | VSL | F | 28 | 25.22 | Overweight | ND | Yes | Housewife | 3 | Bus | Less than an hour | Yes |
| 46 | ATB | HSJL | F | 45 | 32.43 | Class I obesity | ND | Yes | Housewife | 4 | Bus | One hour | Not |
| 47 | ATB | HSJL | F | 22 | 22.35 | Normal | ND | Yes | Student | 1 | Bus | One hour | Not |
| 48 | ATB | HSJL | M | 61 | 21.97 | Normal | ND | Yes | Dependent | 3 | Bus | Less than an hour | Not |
| 49 | ATB | HSJL | M | 48 | 26.08 | Overweight | ND | Yes | Dependent | 4 | Own car | Less than an hour | Not |
| 50 | ATB | HSJL | M | 30 | 25.15 | Overweight | ND | Yes | Independent | 4 | Own car | Less than an hour | Not |
| 51 | ATB | HSJL | F | 68 | 28.35 | Overweight | Negative | Yes | Dependent | 2 | Own car | Less than an hour | Yes |
| 52 | ATB | HSJL | F | 41 | 26.56 | Overweight | ND | Not | Housewife | 4 | Bus | Less than an hour | Yes |
| 53 | ATB | HSJL | M | 44 | 24.16 | Normal | ND | Yes | Independent | 6 | Bus | Less than an hour | Yes |
| 54 | ATB | VSL | F | 31 | 23.44 | Normal | ND | Not | Dependent | 13 | Mototaxi | Three hours | Yes |
| 55 | ATB | VSL | F | 55 | 21.79 | Normal | ND | Yes | Independent | 4 | Bus | One hour | Yes |
| 56 | ATB | VSL | M | 23 | 21.55 | Normal | Positive | Yes | Casual labor | 8 | Bus | Three hours | Not |

HC: Healthy control; LTBI: Latent TB; ATB: Active TB
HSJLH: San Juan de Lurigancho Hospital; VSL: Villa San Luis Center
F: Female; M: Male; BMI: Body Mass Index; TST: Tuberculin skin test; BCG: Bacillus Calmette-Guérin
ND: Not determined.

**Supplementary Table S2**. Cytokine concentration in QFT-Plus TB1 tube from patients with LTBI, ATB and HC

| | QFT-Plus TB1 tube | | | | | | | | | |
|---|---|---|---|---|---|---|---|---|---|---|
| | LTBI | | | ATB | | | HC | | | |
| | **n** | **Media ±DE** | **Mediana (RIQ)** | **n** | **Media ±DE** | **Mediana (RIQ)** | **n** | **Media ±DE** | **Mediana (RIQ)** | **p value †** |
| CD40L | 10 | 145.74 ±226.8 | 74.1 (45.4 - 104.5) | 8 | 158.65 ±175.2 | 115.9 (57.6 - 150.8) | 9 | 210.2 ±232.9 | 166.4 (94.7 - 192.8) | 0.248 |
| EGF | 6 | 80.6 ±100 | 34.7 (7 - 148.7) | 2 | 29.65 ±26.9 | 29.6 (10.6 - 48.7) | 4 | 93.27 ±138.8 | 30.6 (18.6 - 167.9) | 0.938 |
| EOTAXIN | 7 | 21.54 ±13 | 20.1 (10.9 - 30.7) | 4 | 45.73 ±41.2 | 40.6 (14.3 - 77.1) | 10 | 47.91 ±44.3 | 46 (8.3 - 75.7) | 0.49 |
| FGF2 | 5 | 22.1 ±11.9 | 20.2 (13.4 - 29.4) | 4 | 40.18 ±60.6 | 13.3 (7.5 - 72.8) | 14 | 33.02 ±40 | 23.9 (18.8 - 29.4) | 0.705 |
| FLT03L | 14 | 3.19 ±1.9 | 3.2 (1.3 - 5.5) | 9 | 4.1 ±3.2 | 3.5 (2.3 - 4.9) | 13 | 1.43 ±1.1 | 1.2 (0.7 - 2.2) | **0.01** |
| Fractalkine | 7 | 38.08 ±12.9 | 41.1 (29.4 - 41.1) | 4 | 64.45 ±69.4 | 37.7 (22.5 - 106.4) | 13 | 67.15 ±75 | 53.3 (18.3 - 76.9) | 0.855 |
| GCSF | 6 | 71.21 ±81.1 | 30.7 (12.5 - 174.2) | 2 | 430.69 ±30.1 | 430.7 (409.4 - 452) | 10 | 233.42 ±174.8 | 202.2 (129.8 - 303.5) | **0.023** |
| GMCSF | 13 | 364.85 ±805.2 | 103.7 (53.9 - 217.3) | 7 | 386.56 ±540.8 | 94.3 (61 - 915.4) | 10 | 109.66 ±113.4 | 42.8 (29.3 - 190.7) | 0.326 |
| GRO | 7 | 256.41 ±267.4 | 114.6 (56.7 - 624.8) | 6 | 2318.92 ±5077.2 | 206 (192.2 - 605) | 18 | 2703.68 ±5397.2 | 425 (118.8 - 983) | 0.374 |
| IFN-α2 | 10 | 22.73 ±14 | 22.3 (11.4 - 31.8) | 8 | 35.69 ±10.5 | 35.5 (28.8 - 43.7) | 15 | 42.62 ±44.3 | 31.8 (17.4 - 44.5) | 0.143 |
| IFN-γ | 16 | 497.84 ±750.8 | 232.8 (35.6 - 699.3) | 11 | 444.9 ±514.4 | 171 (29.1 - 1009.1) | 8 | 15.66 ±11.5 | 11.6 (8.9 - 23.1) | **0.013** |
| IL1α | 3 | 655.69 ±1090.7 | 49.2 (3 - 1914.9) | 4 | 280.87 ±155.4 | 336.8 (176.6 - 385.1) | 6 | 308.65 ±348.3 | 196.1 (14.9 - 594.9) | 0.841 |
| IL1β | 7 | 218.31 ±293.8 | 52.4 (34 - 517.4) | 6 | 256.82 ±183.6 | 291.4 (53.3 - 404.4) | 9 | 171.74 ±149.8 | 175.5 (46.7 - 230) | 0.577 |
| IL1RA | 17 | 1075.36 ±1073.8 | 795 (203.9 - 1715) | 11 | 810.95 ±801.9 | 613.4 (221.7 - 1275.3) | 21 | 103.97 ±73.3 | 88.7 (51.1 - 163.8) | **<0.001** |
| IL2 | 16 | 377.18 ±593.9 | 184 (17.7 - 528) | 9 | 309.05 ±246.5 | 267.6 (119 - 456.8) | 13 | 4.06 ±4.1 | 2.4 (1.5 - 4.9) | **<0.001** |
| IL3 | 7 | 5.51 ±8 | 1 (0.5 - 13.4) | 5 | 1.81 ±1.2 | 1.6 (0.8 - 2.8) | 6 | 1.43 ±0.8 | 1.6 (1.5 - 1.6) | 0.923 |
| IL4 | 11 | 2.28 ±1.7 | 1.5 (0.8 - 3.9) | 9 | 3.6 ±3.5 | 2.2 (1.3 - 5) | 14 | 1.79 ±1.8 | 1.2 (0.7 - 1.6) | 0.361 |
| IL5 | 15 | 7.95 ±10.2 | 2.9 (0.7 - 11.1) | 9 | 31.48 ±76.8 | 4.8 (0.2 - 4.9) | 12 | 0.54 ±0.4 | 0.4 (0.2 - 0.9) | **0.013** |
| IL6 | 5 | 1033.47 ±1139.1 | 452.1 (202.8 - 1923.7) | 4 | 1305.93 ±765.9 | 1240.6 (658.5 - 1953.4) | 14 | 1309.92 ±1281.1 | 890.8 (510.8 - 1781.8) | 0.674 |

| | | | | | | | | | | |
|---|---|---|---|---|---|---|---|---|---|---|
| IL7 | 8 | 1.15 ±0.5 | 1.1 (0.8 - 1.5) | 5 | 1.65 ±0.9 | 1.7 (0.9 - 1.9) | 12 | 1.36 ±1.2 | 1 (0.5 - 1.9) | 0.664 |
| IL8 | 10 | 2112.15 ±2510.6 | 741.1 (376.8 - 5075.8) | 4 | 1135.08 ±1287.6 | 744.3 (221.1 - 2049.1) | 12 | 548.68 ±596.1 | 321.2 (115.1 - 932.7) | 0.147 |
| IL9 | 8 | 13.73 ±13.9 | 8.7 (1.5 - 27.7) | 6 | 18.03 ±14 | 16.4 (5.6 - 27.7) | 8 | 19.15 ±18.4 | 14 (8 - 25.6) | 0.645 |
| IL10 | 6 | 4.84 ±5 | 2.6 (1.3 - 10.2) | 5 | 2.72 ±1.6 | 2.1 (1.3 - 4.3) | 8 | 4.33 ±4.4 | 2.6 (2 - 5.1) | 0.701 |
| IL12P40 | 10 | 39.44 ±24.9 | 30.3 (20.4 - 60) | 8 | 67.23 ±65.1 | 35.3 (23.5 - 108.7) | 11 | 39.96 ±42.8 | 28.5 (18.5 - 40) | 0.623 |
| IL12P70 | 9 | 3.79 ±1.5 | 3.6 (2.7 - 4.8) | 5 | 13.26 ±18.9 | 5.7 (5 - 7.6) | 8 | 10.85 ±14.2 | 5 (3.6 - 11.5) | 0.176 |
| IL13 | 11 | 26.43 ±21.7 | 13.1 (8.3 - 45.6) | 7 | 48.51 ±59.5 | 25 (18 - 44.8) | 14 | 19.57 ±18.1 | 13.9 (8.8 - 18.4) | 0.23 |
| IL15 | 9 | 12.91 ±16.5 | 7.8 (4.9 - 11.4) | 8 | 10.84 ±7.4 | 8.4 (5.7 - 15.3) | 14 | 11.94 ±15.1 | 9.1 (3.3 - 14.2) | 0.937 |
| IL17α | 6 | 16.26 ±18.9 | 10.1 (4.6 - 14.4) | 4 | 15.8 ±8.6 | 15.7 (8.5 - 23.1) | 13 | 8.97 ±9 | 6.9 (3.7 - 10.1) | 0.162 |
| IP10 | 13 | 15764.94 ±16032.5 | 4549.5 (1057.5 - 31720.2) | 5 | 3071.9 ±4062.6 | 1680.2 (719.9 - 2429.5) | 18 | 1869.04 ±5800.4 | 260.4 (36.6 - 560.6) | **0.002** |
| MCP1 | 11 | 8369.89 ±15239.6 | 1702.5 (385.6 - 4210.3) | 5 | 3211.91 ±2880 | 1543.9 (1241.8 - 4658.8) | 20 | 6060.78 ±10523.7 | 1887.7 (1118.8 - 3128.8) | 0.95 |
| MCP3 | 16 | 1794.49 ±1938.3 | 1093.6 (547.3 - 2075.4) | 11 | 10219.54 ±27845.9 | 1135.7 (667.4 - 2866.7) | 19 | 785.09 ±883.6 | 337.4 (185.2 - 1211.4) | 0.063 |
| MDC | 9 | 68.09 ±59.4 | 33.3 (24.3 - 120.4) | 10 | 115.08 ±117.1 | 68.4 (30.9 - 212.9) | 17 | 77.63 ±66.2 | 43.9 (30.8 - 119.8) | 0.597 |
| MIP1α | 8 | 5859.89 ±15521.2 | 410 (130.6 - 757.2) | 6 | 801.75 ±716.1 | 529.3 (204.9 - 1689.2) | 15 | 6284.78 ±12049.4 | 570.4 (375.3 - 1466.5) | 0.46 |
| MIP1β | 16 | 781.92 ±818.6 | 363.5 (205.1 - 1475.4) | 10 | 699.86 ±855.4 | 333.3 (229.6 - 814) | 15 | 769.04 ±1000.2 | 485.9 (94.5 - 908) | 0.993 |
| TGFα | 16 | 4.61 ±3 | 3.6 (2.8 - 7.5) | 9 | 7.72 ±5.1 | 6.9 (4.8 - 11) | 13 | 5.45 ±8.7 | 3 (1.2 - 4.2) | 0.137 |
| TNFα | 13 | 363.58 ±438.2 | 189.1 (143 - 349.4) | 6 | 499.4 ±504 | 258.3 (178.6 - 909.9) | 15 | 344.87 ±412.9 | 155.1 (53.1 - 685.5) | 0.461 |
| TNFβ | 6 | 4.1 ±2.5 | 4.5 (1.7 - 5.8) | 7 | 4.98 ±2.3 | 4.1 (3.4 - 8) | 15 | 7.48 ±9.3 | 4 (1.7 - 10.3) | 0.804 |
| VEGFα | 2 | 2.03 ±0.8 | 2 (1.4 - 2.6) | 2 | 13.9 ±2.5 | 13.9 (12.2 - 15.6) | 9 | 84.17 ±106.3 | 48.3 (15.7 - 109.4) | 0.134 |

†: Kruskal–Wallis test

HC: Healthy control; LTBI: Latent TB; ATB: Active TB

SD: Standard Deviation; IQ: Interquartile Range; QFT-Plus: QuantiFERON-Plus

n: Sample size

**Supplementary Table S3.** Cytokine concentration in QFT-Plus TB2 tube from patients with LTBI, ATB and HC

| | **QFT-Plus TB2 tube** | | | | | | | | | |
|---|---|---|---|---|---|---|---|---|---|---|
| | **LTBI** | | | **ATB** | | | **HC** | | | |
| | **n** | **Media ±DE** | **Mediana (RIQ)** | **n** | **Media ±DE** | **Mediana (RIQ)** | **n** | **Media ±DE** | **Mediana (RIQ)** | **p value †** |
| CD40L | 11 | 137.82 ±151.6 | 11 (137.8 - 151.6) | 5 | 339.07 ±514.8 | 5 (339.1 - 514.8) | 14 | 298.51 ±551 | 14 (298.5 - 551) | 0.652 |
| EGF | 5 | 53.67 ±29.1 | 5 (53.7 - 29.1) | 2 | 20.27 ±3 | 2 (20.3 - 3) | 6 | 101.22 ±99.3 | 6 (101.2 - 99.3) | 0.245 |
| EOTAXIN | 6 | 30.37 ±25.8 | 6 (30.4 - 25.8) | 5 | 56.84 ±51.2 | 5 (56.8 - 51.2) | 14 | 46.18 ±71.2 | 14 (46.2 - 71.2) | 0.503 |
| FGF2 | 6 | 28.4 ±17.8 | 6 (28.4 - 17.8) | 4 | 81.12 ±124.2 | 4 (81.1 - 124.2) | 10 | 71.46 ±141.7 | 10 (71.5 - 141.7) | 0.901 |
| FLT03L | 13 | 3.54 ±2.4 | 13 (3.5 - 2.4) | 10 | 4.1 ±3.1 | 10 (4.1 - 3.1) | 13 | 2.86 ±5.6 | 13 (2.9 - 5.6) | 0.053 |
| Fractalkine | 7 | 53.03 ±51.2 | 7 (53 - 51.2) | 6 | 144.52 ±253.7 | 6 (144.5 - 253.7) | 12 | 93.99 ±185 | 12 (94 - 185) | 0.933 |
| GCSF | 5 | 116.67 ±89.6 | 5 (116.7 - 89.6) | 2 | 495.18 ±229.9 | 2 (495.2 - 229.9) | 11 | 241.73 ±304 | 11 (241.7 - 304) | 0.162 |
| GMCSF | 13 | 317.71 ±618.5 | 13 (317.7 - 618.5) | 7 | 440.21 ±763.1 | 7 (440.2 - 763.1) | 11 | 157.99 ±343.4 | 11 (158 - 343.4) | **0.025** |
| GRO | 8 | 273.98 ±369.7 | 8 (274 - 369.7) | 6 | 3037.17 ±6579.1 | 6 (3037.2 - 6579.1) | 13 | 6569.56 ±8767.8 | 13 (6569.6 - 8767.8) | 0.18 |
| IFN-α2 | 9 | 26.63 ±16 | 9 (26.6 - 16) | 6 | 49.74 ±35.6 | 6 (49.7 - 35.6) | 15 | 45.02 ±44.1 | 15 (45 - 44.1) | 0.268 |
| IFN-γ | 15 | 496.23 ±630.5 | 15 (496.2 - 630.5) | 10 | 474.22 ±460.4 | 10 (474.2 - 460.4) | 9 | 25.83 ±36.6 | 9 (25.8 - 36.6) | **0.002** |
| IL1α | 4 | 171.23 ±324.9 | 4 (171.2 - 324.9) | 2 | 99.93 ±84.3 | 2 (99.9 - 84.3) | 4 | 580.95 ±920.3 | 4 (581 - 920.3) | 0.646 |
| IL1β | 4 | 199.21 ±207.8 | 4 (199.2 - 207.8) | 3 | 336.96 ±253.9 | 3 (337 - 253.9) | 9 | 204.66 ±378.1 | 9 (204.7 - 378.1) | 0.156 |
| IL1RA | 17 | 934.99 ±917.8 | 17 (935 - 917.8) | 11 | 1071.98 ±1265.4 | 11 (1072 - 1265.4) | 22 | 90.52 ±102 | 22 (90.5 - 102) | **<0.001** |
| IL2 | 17 | 305.58 ±434.4 | 17 (305.6 - 434.4) | 9 | 315.15 ±272.1 | 9 (315.2 - 272.1) | 12 | 9.23 ±15.7 | 12 (9.2 - 15.7) | **<0.001** |
| IL3 | 6 | 7.14 ±8 | 6 (7.1 - 8) | 5 | 2.33 ±2.9 | 5 (2.3 - 2.9) | 6 | 1.33 ±0.9 | 6 (1.3 - 0.9) | 0.145 |
| IL4 | 8 | 2.35 ±1.7 | 8 (2.3 - 1.7) | 6 | 4.35 ±5.3 | 6 (4.3 - 5.3) | 14 | 3.67 ±8.5 | 14 (3.7 - 8.5) | 0.316 |
| IL5 | 15 | 6.1 ±8 | 15 (6.1 - 8) | 8 | 41.81 ±102.8 | 8 (41.8 - 102.8) | 13 | 3.57 ±11.4 | 13 (3.6 - 11.4) | **0.002** |

| | | | | | | | | | | |
|---|---|---|---|---|---|---|---|---|---|---|
| IL6 | 6 | 556.69 ±824.6 | 6 (556.7 - 824.6) | 4 | 819.25 ±510.6 | 4 (819.3 - 510.6) | 11 | 2242.73 ±4184.8 | 11 (2242.7 - 4184.8) | 0.316 |
| IL7 | 5 | 1.68 ±0.9 | 5 (1.7 - 0.9) | 6 | 2.14 ±2.4 | 6 (2.1 - 2.4) | 10 | 1.84 ±2.8 | 10 (1.8 - 2.8) | 0.551 |
| IL8 | 10 | 2035.28 ±1625.8 | 10 (2035.3 - 1625.8) | 5 | 887.48 ±1226.3 | 5 (887.5 - 1226.3) | 17 | 538.62 ±616.7 | 17 (538.6 - 616.7) | 0.06 |
| IL9 | 4 | 15.16 ±13.7 | 4 (15.2 - 13.7) | 4 | 23.52 ±14.1 | 4 (23.5 - 14.1) | 13 | 13.19 ±9.3 | 13 (13.2 - 9.3) | 0.319 |
| IL10 | 5 | 9.8 ±10.7 | 5 (9.8 - 10.7) | 4 | 5.21 ±5 | 4 (5.2 - 5) | 7 | 8.66 ±11.9 | 7 (8.7 - 11.9) | 0.568 |
| IL12P40 | 10 | 43.32 ±29.7 | 10 (43.3 - 29.7) | 5 | 155.35 ±235 | 5 (155.4 - 235) | 15 | 54.83 ±115.8 | 15 (54.8 - 115.8) | 0.119 |
| IL12P70 | 8 | 5.61 ±3.6 | 8 (5.6 - 3.6) | 5 | 25.86 ±47 | 5 (25.9 - 47) | 9 | 29.15 ±71.7 | 9 (29.2 - 71.7) | 0.956 |
| IL13 | 10 | 33.18 ±23.6 | 10 (33.2 - 23.6) | 7 | 58.8 ±85 | 7 (58.8 - 85) | 14 | 31.66 ±62.1 | 14 (31.7 - 62.1) | 0.168 |
| IL15 | 8 | 15.06 ±12.1 | 8 (15.1 - 12.1) | 6 | 12.57 ±9.6 | 6 (12.6 - 9.6) | 14 | 13.02 ±8.5 | 14 (13 - 8.5) | 0.953 |
| IL17α | 7 | 14.2 ±12.6 | 7 (14.2 - 12.6) | 5 | 22.35 ±20.1 | 5 (22.3 - 20.1) | 11 | 17.56 ±24.4 | 11 (17.6 - 24.4) | 0.492 |
| IP10 | 11 | 13177.59 ±15782.3 | 11 (13177.6 - 15782.3) | 5 | 5660.42 ±5314.5 | 5 (5660.4 - 5314.5) | 17 | 2074.56 ±6550.4 | 17 (2074.6 - 6550.4) | **0.001** |
| MCP1 | 12 | 2062.77 ±2017.8 | 12 (2062.8 - 2017.8) | 8 | 1383.34 ±950.3 | 8 (1383.3 - 950.3) | 19 | 6986.64 ±12370.7 | 19 (6986.6 - 12370.7) | 0.799 |
| MCP3 | 17 | 1182.27 ±1670.5 | 17 (1182.3 - 1670.5) | 11 | 13733.08 ±39826.8 | 11 (13733.1 - 39826.8) | 17 | 1052.24 ±1268.6 | 17 (1052.2 - 1268.6) | 0.654 |
| MDC | 7 | 85.81 ±75.1 | 7 (85.8 - 75.1) | 7 | 94.15 ±115.3 | 7 (94.1 - 115.3) | 14 | 55.36 ±46.8 | 14 (55.4 - 46.8) | 0.692 |
| MIP1α | 8 | 5227.03 ±13193.7 | 8 (5227 - 13193.7) | 8 | 1833.22 ±4129.6 | 8 (1833.2 - 4129.6) | 14 | 6295.5 ±14840.1 | 14 (6295.5 - 14840.1) | 0.207 |
| MIP1β | 12 | 1007.13 ±860.6 | 12 (1007.1 - 860.6) | 10 | 718.34 ±683.4 | 10 (718.3 - 683.4) | 13 | 428.82 ±503.6 | 13 (428.8 - 503.6) | 0.097 |
| TGFα | 13 | 5.25 ±2.5 | 13 (5.3 - 2.5) | 9 | 11.22 ±11 | 9 (11.2 - 11) | 13 | 16.48 ±49.3 | 13 (16.5 - 49.3) | 0.111 |
| TNFα | 12 | 261.83 ±277.8 | 12 (261.8 - 277.8) | 6 | 313.22 ±232.9 | 6 (313.2 - 232.9) | 17 | 176.95 ±228.6 | 17 (176.9 - 228.6) | 0.463 |
| TNFβ | 7 | 5.66 ±5.2 | 7 (5.7 - 5.2) | 6 | 10.58 ±18.9 | 6 (10.6 - 18.9) | 13 | 16.08 ±45.1 | 13 (16.1 - 45.1) | 0.858 |
| VEGFα | 0 | * | * | 1 | 14.44 ±0 | 1 (14.4 - 0) | 9 | 68.81 ±93.9 | 9 (68.8 - 93.9) | 0.600 |

†: Kruskal–Wallis test
HC: Healthy control; LTBI: Latent TB; ATB: Active TB
SD: Standard Deviation; IQR: Interquartile Range; QFT-Plus: QuantiFERON-Plus
n: Sample size
*: Not determined

**Supplementary Table S4.** Studies related to IP-10 and Tuberculosis

| First autor (year) | Article | Population / setting | N (cases / controls or groups) | Specimen / assay | Comparison groups | AUC | Sensitivity | Specificity |
|---|---|---|---|---|---|---|---|---|
| Qiu X, et al. (2019) | Diagnostic accuracy of interferon-gamma-induced protein 10 for differentiating active tuberculosis from latent tuberculosis: A meta-analysis | Meta-analysis | 706 participants | Various | ATB vs LTBI | 0.86 | 0.72 | 0.83 |
| Petrone L, et al. (2018) | Evaluation of IP-10 in Quantiferon-Plus as biomarker for the diagnosis of latent tuberculosis infection | Hospital / diagnostic setting | 36 ATB / 31 LTBI / 16 HC | Plasma, ELISA (QFT-Plus supernatant) | ATB vs HC | (ROC performed) | 0.86 | 0.94 |
| Blauenfeldt T, et al. (2020) | Diagnostic Accuracy of Interferon Gamma-Induced Protein 10 mRNA Release Assay for Tuberculosis | Case-control clinical study | 89 ATB / 99 HC | QFT tubes, IP-10 mRNA (molecular) | ATB vs HC | 0.98 | 0.87 | 0.98 |
| Zhang W. (2019) | IP-10 for the diagnosis of tuberculosis in children: Protocol for a systematic review and meta-analysis | Pediatric cohorts | variable | Serum / QFT supernatant | ATB vs LTBI / HC | 0.8–0.9 | Variable | Variable |

HC: Healthy control; LTBI: Latent TB; ATB: Active TB
QFT-Plus: QuantiFERON-Plus
